# Outcome of transarterial radioembolization in patients with hepatocellular carcinoma as a first-line interventional therapy and after a previous transarterial chemoembolization

**DOI:** 10.3389/fradi.2024.1346550

**Published:** 2024-02-20

**Authors:** Julia Wagenpfeil, Patrick Arthur Kupczyk, Philipp Bruners, Robert Siepmann, Emelie Guendel, Julian Alexander Luetkens, Alexander Isaak, Carsten Meyer, Fabian Kuetting, Claus Christian Pieper, Ulrike Irmgard Attenberger, Daniel Kuetting

**Affiliations:** ^1^ Department of Diagnostic and Interventional Radiology, University Hospital of Bonn, Bonn, Germany; ^2^ Center for Integrated Oncology (CIO), Aachen-Bonn-Cologne-Duesseldorf, Germany; ^3^ Department of Diagnostic and Interventional Radiology, University Hospital of Aachen, Aachen, Germany

**Keywords:** hepatocellular carcinoma, transarterial radioembolization, transarterial chemoembolization, interventional therapy, Barcelona clinic liver cancer staging system

## Abstract

**Purpose:**

Due to a lack of data, there is an ongoing debate regarding the optimal frontline interventional therapy for unresectable hepatocellular carcinoma (HCC). The aim of the study is to compare the results of transarterial radioembolization (TARE) as the first-line therapy and as a subsequent therapy following prior transarterial chemoembolization (TACE) in these patients.

**Methods:**

A total of 83 patients were evaluated, with 38 patients having undergone at least one TACE session prior to TARE [27 male; mean age 67.2 years; 68.4% stage Barcelona clinic liver cancer (BCLC) B, 31.6% BCLC C]; 45 patients underwent primary TARE (33 male; mean age 69.9 years; 40% BCLC B, 58% BCLC C). Clinical [age, gender, BCLC stage, activity in gigabecquerel (GBq), Child–Pugh status, portal vein thrombosis, tumor volume] and procedural [overall survival (OS), local tumor control (LTC), and progression-free survival (PFS)] data were compared. A regression analysis was performed to evaluate OS, LTC, and PFS.

**Results:**

No differences were found in OS (95% CI: 1.12, *P *= 0.289), LTC (95% CI: 0.003, *P *= 0.95), and PFS (95% CI: 0.4, *P *= 0.525). The regression analysis revealed a relationship between Child–Pugh score (*P *= 0.005), size of HCC lesions (>10 cm) (*P *= 0.022), and OS; neither prior TACE (Child–Pugh B patients; 95% CI: 0.120, *P *= 0.729) nor number of lesions (>10; 95% CI: 2.930, *P *= 0.087) correlated with OS.

**Conclusion:**

Prior TACE does not affect the outcome of TARE in unresectable HCC.

## Introduction

Hepatocellular carcinoma (HCC), the third leading cause of cancer-related mortality, constitutes 75%–85% of primary liver malignancies (1, 2). The main risk factors for HCC vary geographically but generally include chronic hepatitis B virus (HBV) and C virus (HCV), as well as alcohol-associated cirrhosis and non-alcoholic steatohepatitis (2–4). The type of therapy depends on several factors, e.g., HCC size and number of lesions, location, portal vein infiltration, and liver function.

Based on the individual Barcelona clinic liver cancer (BCLC) stage, the treatment of choice varies from resection/local ablation to chemotherapy/immunotherapy.

However, in most cases, HCC is diagnosed in advanced stages, highlighting the need for effective systemic therapies.

Patients with locally advanced tumor disease with vascular infiltration, especially in the presence of extrahepatic manifestations, have shown significant progress in treatment. One example is the use of oral multikinase inhibitors such as sorafenib, which inhibits multiple receptor tyrosine kinases in addition to intracellular kinases (Raf1/B-Raf); its efficacy and safety have been demonstrated in Phase II/III studies (5).

In recent years in particular, rapid progress has also been achieved in the field of immunotherapy with the approval of checkpoint inhibitors for the treatment of advanced HCC. Particularly noteworthy results were achieved via a combination therapy of a PDL-1 inhibitor and a VEGF antibody (atezolizumab and bevacizumab) (6).

For the intermediate-stage (BCLC B) patients, transarterial chemoembolization (TACE) is the preferred option (7, 8). Transarterial radioembolization (TARE), frequently performed as a second-line therapy in case of TACE failure, is preferred by some institutions as the primary treatment in BCLC B (9, 10). Although both TACE and TARE in HCC have been extensively studied, there is a dearth of data regarding the potential impact of prior TACE therapy on the outcome of TARE treatment.

The present study aims to compare the outcome of TARE treatment in patients with and without prior TACE.

## Materials and methods

### Patient cohort

In this multicenter retrospective study, patients with unresectable, non-metastasized hepatocellular carcinoma treated with TACE and TARE (Group A) or solely TARE (Group B) between February 2011 and July 2019 were included. The inclusion criteria were non-metastatic HCC with or without portal vein thrombosis, Child–Pugh stage A/B, BCLC stages A–C, Eastern Cooperative Oncology Group (ECOG) Stage 0, no prior intra-arterial treatment, and availability of procedural, clinical, and follow-up data. Patients who had received TACE therapy after TARE therapy were excluded from the study (Table 1).

**Table 1:** PRISMA flow chart.

“ 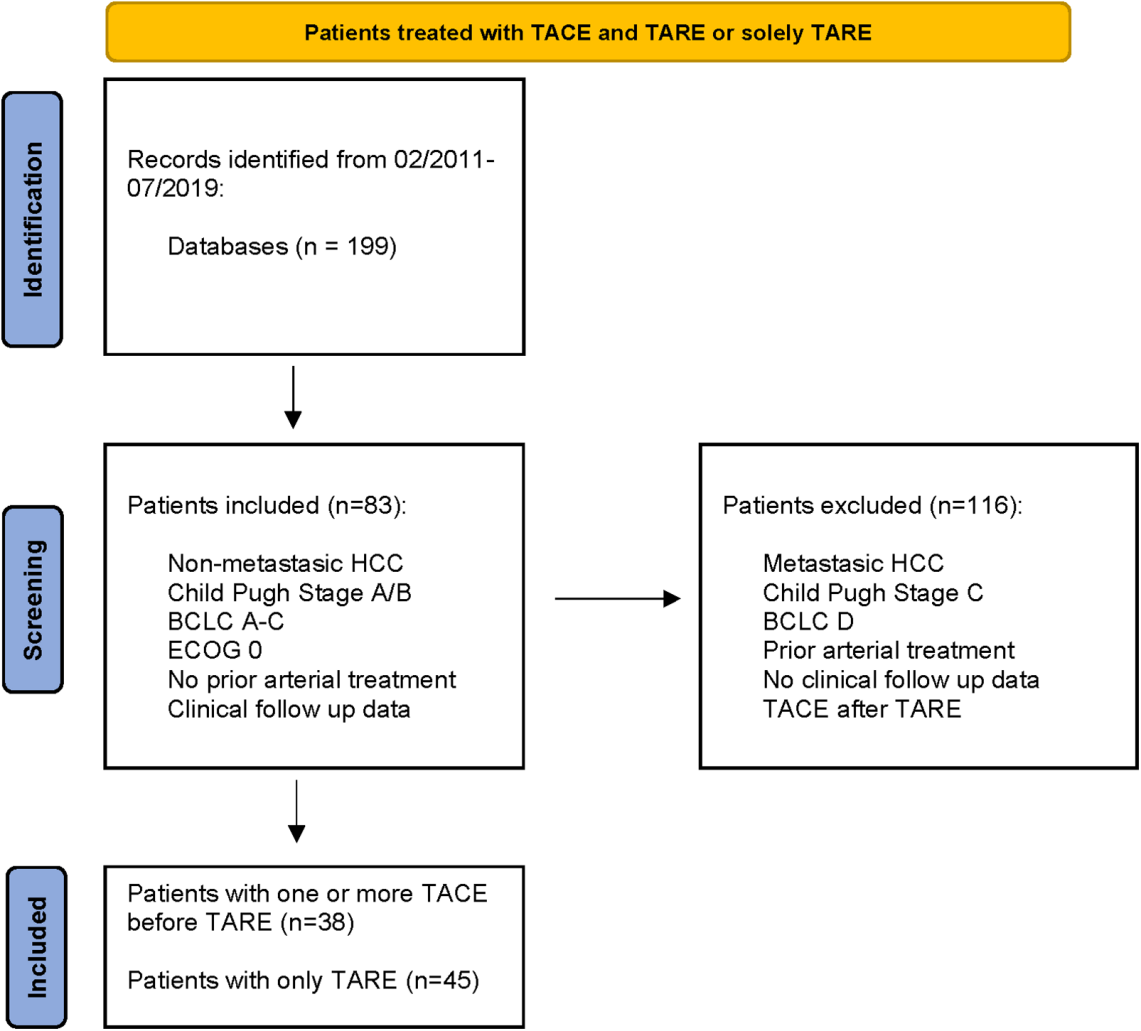 ”

A total of 199 patients were reviewed, and 116 patients were excluded. Overall, 83 patients were eligible. Thirty-eight consecutive patients initially underwent one or more TACE sessions before receiving TARE therapy; the control group consisted of 45 patients who received TARE without receiving prior chemoembolization treatment. Clinical [age, gender, BCLC stage, activity in gigabecquerel (GBq), Child–Pugh status, portal vein thrombosis, and tumor volume] and procedural [overall survival (OS), local tumor control (LTC), and progression-free survival (PFS)] data were analyzed and compared between the two groups as previously described (9). The therapy indication was confirmed by an interdisciplinary tumor board. The institutional ethics committee approved data analysis with a waiver for additional informed patient consent.

### Follow-up

The baseline was established from the most recent computed tomography (CT) or magnetic resonance imaging (MRI) conducted prior to TARE. All patients underwent continuous follow-up, including clinical visits, PET CT, and liver MRI.

### Definitions

Tumor response assessment was defined using the Modified Response Evaluation Criteria in Solid Tumors (mRECIST) (11). LTC was defined as the time until the progression of any tumor lesion within a treated segment following TARE. PFS was defined as the time between TARE and intra- or extrahepatic tumor progression. OS was determined as the time period from the first treatment date to either the date of death or the last date of follow-up.

### Statistical analysis

OS, LTC, and PFS were assessed in all patients and compared between the two groups using the Chi-square test. Furthermore, subgroup analyses for OS and LTC were performed for BCLC stage B patients and compared between the two groups. Patients lost to follow-up were censored (contingency tables for proportional distribution). *P*-values < 0.05 were considered significant. A Cox regression analysis was conducted with covariates including age, BCLC stage, Child–Pugh score, activity in GBq, previous TACE seasons (number of TACE), and the size and number of HCC lesions to evaluate the impact on the outcome.

## Results

### Overall cohort characteristics

A total of 83 patients were included, and 38 patients received at least one TACE session prior to TARE therapy (Group A; 11 female, 27 male; mean age 67.2 years). Ten patients underwent one TACE therapy prior to TARE, 10 patients received two TACE treatments, seven patients received three treatments, five patients underwent four treatments, five patients received five treatments, and one patient underwent seven cycles of TACE. Partial portal vein thrombosis was detected in four patients (10.5%), and bilobar HCC manifestation was identified in 34 patients (89.5%). A total of 29 patients (73.3%) were classified as Child–Pugh A, while nine patients (23.7%) were classified as Child–Pugh B. None of the patients fell under the classification of Child–Pugh C. A total of 26 patients (68.4%) were graded stage BCLC B, and 12 patients (31.6%) were graded stage BCLC C (see Table 2 for details).

**Table 2:** Patient characteristics.

TACE + TARE		TARE	
(Group A)		(Group B)	
Variable	Value (%)	Variable	Value (%)
Number	38	Number	45
Sex		Sex	
Male	27 (71)	Male	33 (73.3)
Female	11 (39.3)	Female	12 (26.7)
Mean age [years ± SD]	67.2 ± 9.5	Mean age [years ± SD]	69.9 ± 9.1
Mean TARE doses GBq	1.08	Mean TARE doses GBq	1.71
Number of TACE			
One	10 (26.3)		
Two	10 (26.3)		
Three	7 (18.4)		
Four	5 (13.1)		
Five	5 (13.1)		
Seven	1 (2.6)		
Portal vein		Portal vein	
Thrombosis/infiltration	4 (10.5)	Thrombosis/infiltration	20 (44.4)
Bilobar manifestation	34 (89.5)	Bilobar manifestation	33 (73.3)
Child–Pugh score		Child–Pugh score	
A	29 (73.3)	A	16 (35.6)
B	9 (23.7)	B	21 (46.7)
C	0	C	0
Incomplete data	0	Incomplete data	8 (17.8)
BCLC		BCLC	
B	26 (68.4)	B	18 (40)
C	12 (31.6)	C	26 (58)

The control group consisted of 45 patients who received TARE therapy without prior TACE (Group B; 12 female, 33 male; mean age 69.9 years). Portal vein thrombosis was detected in 20 patients (44.4%), and bilobar HCC manifestation was observed in 33 patients (73.3%). Sixteen patients (35.6%) were classified as Child–Pugh A, 21 patients (46.7%) were classified as Child–Pugh B, and none of the patients were categorized as Child–Pugh C. Laboratory results were incomplete in eight patients (17.8%). Eighteen patients (40%) were graded as stage BCLC B, 26 patients (58%) were graded as stage BCLC C, and BCLC stage could not be defined (2%) in one patient (see Table 2 for details).

### Statistical analysis

**Figure 1:**
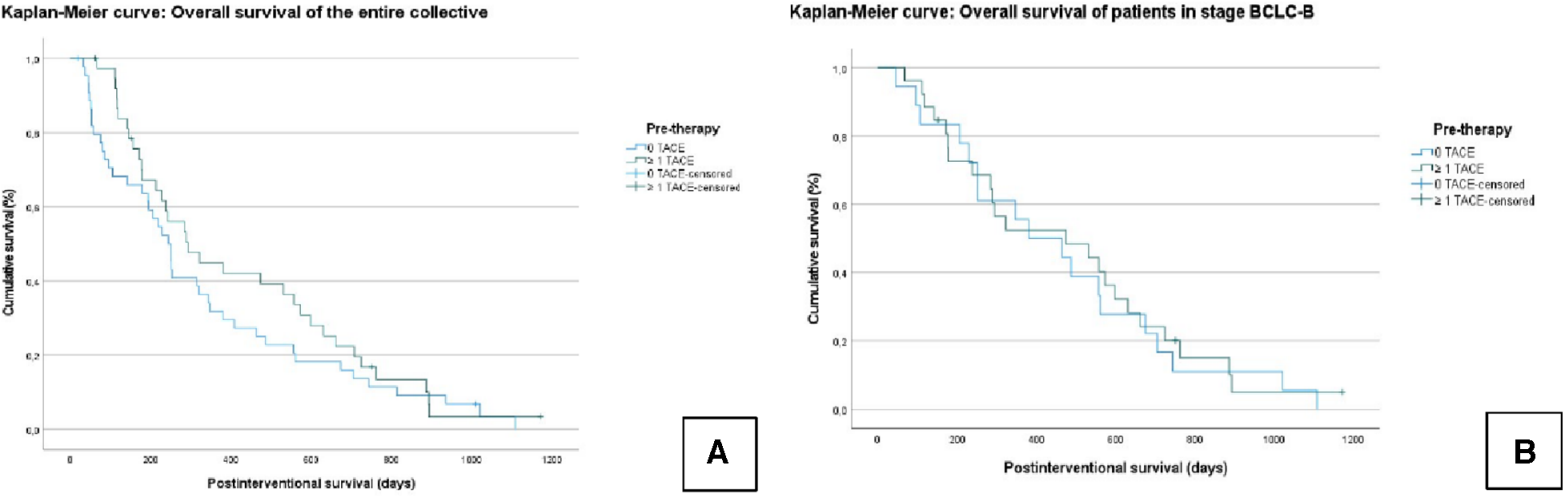
Kaplan–Meier curves: overall survival (OS) of the entire collective (**A**); overall survival of patients who graduated stage B (BCLC) (**B**). No significant difference is seen between the TACE/TARE and TARE-only groups.

In the entire collective, the median OS was 375.7 ± 34 days (95% CI = 308–442). In Group A, the median OS was 421.6 ± 49.7 days (95% CI = 324–518). In Group B, the median OS was 334.8 ± 45.9 days (95% CI = 244–424); *χ*²* *= 1.126; *P* = 0.289 (Figure 1).

In the subgroup analysis, the median OS in all BCLC B patients was 468.2 ± 46 days (95% CI = 377–558). In Group A, the median OS was 473.9 ± 60.6 days (95% CI = 355–592), and in Group B, the median OS was 457.3 ± 72.9 days (95% CI = 315–598); *χ*² = 0.123; *P* = 0.726 (Figure 1).

**Figure 2:**
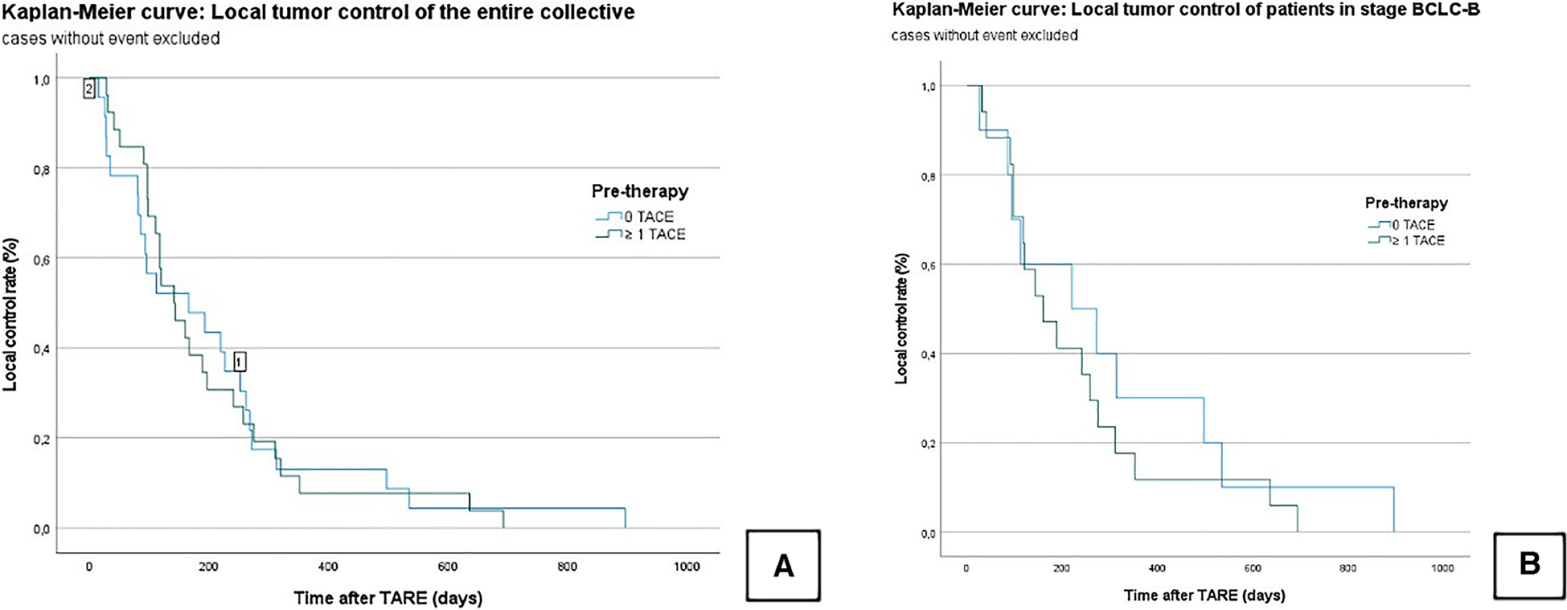
Kaplan–Meier curves: local tumor control of the censored entire collective (**A**); local tumor control of censored patients who graduated stage B (BCLC) (**B**). No significant difference is seen between the TACE/TARE and TARE-only group.

The LTC in the entire collective was 201.4 ± 26.2 days (95% CI = 150–252). In Group A, the LTC was 195.6 ± 32.3 days [95% confidence interval (CI) = 132–258], and in Group B, the LTC was 208 ± 43 days (95% CI = 123–292); *χ*² = 0.003; *P* = 0.956 (Figure 2).

In the subgroup analysis, the median LTC in all BCLC B patients was 255.7 ± 42.5 days (95% CI = 172–338). In Group A, the median LTC was 305.1 ± 85.3 days (95% CI = 137–472), and in Group B, it was 226.6 ± 45.8 days (95% CI = 136–316); *χ*² = 0.568; *P* = 0.451 (Figure 2).

**Figure 3:**
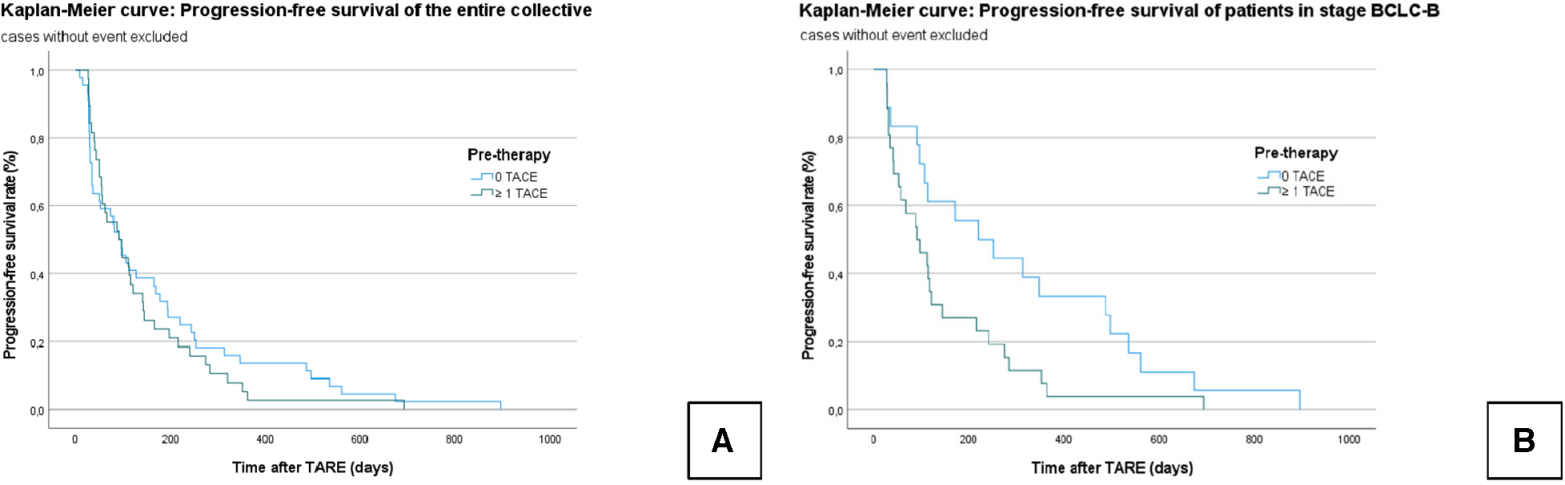
Kaplan–Meier curves: progression-free survival of the censored entire collective (**A**): no significant difference is seen between the TACE/TARE and TARE-only groups. Progression-free survival of censored patients who graduated stage B (BCLC) (**B**): A significant difference is seen between the TACE/TARE and TARE-only groups.

In the entire collective, the median PFS was 154.9 ± 19 days (95% CI = 117–192). In Group A, the median PFS was 134.3 ± 21.7 days (95% CI = 92–177), and in Group B, it was 172.8 ± 30.4 days (95% CI = 113–232); *χ*² = 0.404; *P* = 0.525 (Figure 3).

In the subgroup analysis, the median PFS in all BCLC B patients was 208.7 ± 32 days (95% CI = 146–271); Group A: 143.7 ± 29.8 days (95% CI = 82–202); Group B: 302.4 ± 59.9 days (95% CI = 185–420); *χ*² = 4.680; *P* = 0.031) (Figure 3).

Cox regression analysis for age (*P *= 0.736), activity in GBq (*P *= 0.805), number of treatments (*P *= 0.308), number of lesions (*P *= 0.916), and lesion size <5 cm (*P *= 0.072) and <10 cm (*P *= 0.257) referred to overall survival did not reach statistical significance. The Child–Pugh score (*P *= 0.005) and size of lesions >10 cm (*P *= 0.022) showed hazard ratios of 2,717 (Child–Pugh score) and 2,505 (size of lesion).

**Figure 4:**
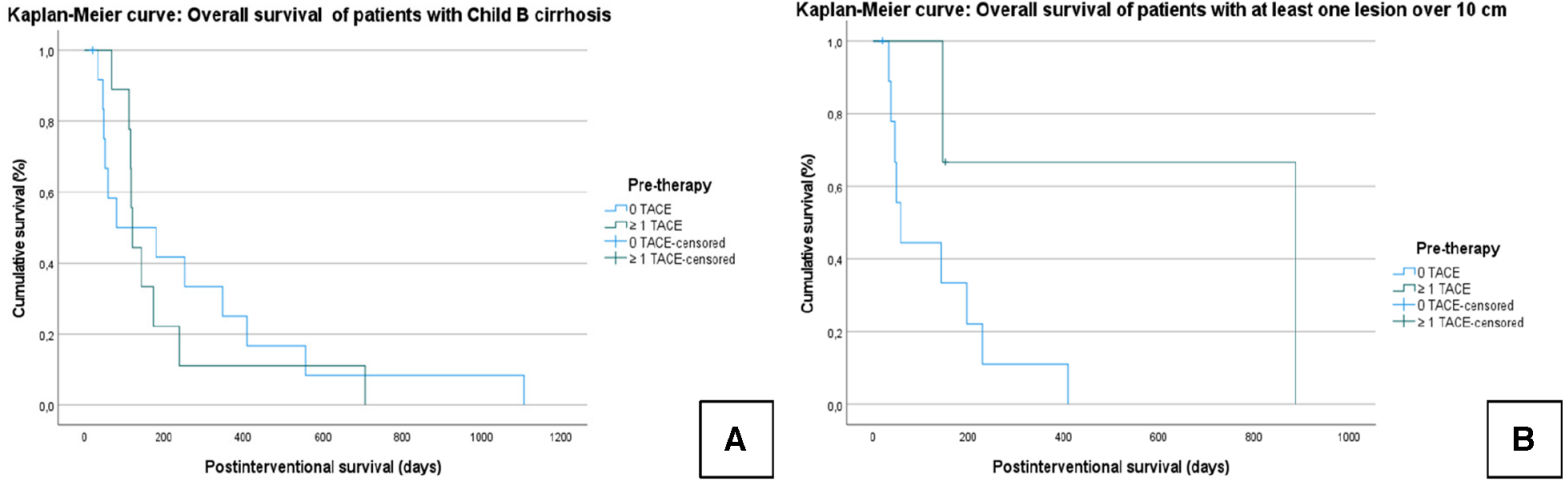
Kaplan–Meier curves: the median overall survival of patients with Child B cirrhosis (**A**) and with at least one lesion over 10 cm (**B**). For both TACE and TARE, the Child–Pugh score and lesion size (>10 cm) had an effect on the outcome; a reduced OS was found in patients with higher tumor burden and reduced liver function. However, the OS in this subgroup did not differ depending on whether a prior TACE had been performed.

In patients classified as Child–Pugh B, the median OS was 236 ± 58.4 days (95% CI = 121.6–350); Group A: 199 ± 65.4 days (95% CI = 70–327); Group B: 263 ± 91 days (95% CI = 85–443); *χ*² = 0.120; *P* = 0.729 (Figure 4A).

In patients with lesions >10 cm, the median OS was 221.6 ± 81 days (95% CI = 62–380); Group A: 639.7 ± 283.6 days (95% CI = 80–1199); Group B: 133 ± 42.4 days (95% CI = 50–216); *χ*² = 2.930; *P* = 0.087 (Figure 4B).

### Outcome

No differences in OS, LTC, and PFS were found between patients receiving TACE before TARE and those receiving only TARE.

The Child–Pugh score and the size of the HCC lesions (>10 cm) correlated with OS; neither previous TACE nor the number of lesions correlated with OS.

## Discussion

This study examined the outcome of TARE in patients with prior TACE treatment and compared it with those without prior embolic therapy for unresectable HCC. The main findings are that the outcomes of TARE do not differ between patients who received TACE prior to radioembolization and those who only received radioembolization in a patient collective with mainly advanced HCC.

For HCC patients who are not eligible for transplantation, local ablation, or resection due to tumor location and/or several tumor lesions (i.e., BCLC stage B patients), transarterial chemoembolization is a validated treatment option (7); TARE is a suitable treatment alternative for unresectable intermediate-stage HCC and even offers further advantages in cases where ablative radioembolization/radiation segmentectomy is possible (12, 13).

Nonetheless, TACE remains the standard of care in intermediate well-defined HCC, due to the lack of randomized controlled trial data proving the superiority of TARE, as well as its substantially higher procedural costs of TARE (9, 14–19).

Thus, in a clinical setting, it is common for patients to receive TARE only after initial TACE failure. Until now, the efficacy of TARE has not been evaluated in patients with prior chemoembolic treatment.

A possible downside of initial TACE may result from macro- and microvascular damage caused by repetitive embolization, potentially reducing the effects of a second-line TARE therapy. However, HCC progression is commonly based on neo-angiogenesis; thus, in a growing or *de novo* HCC lesion, new or recanalized feeding vessels are to be expected to facilitate further embolic therapy (16, 20). This is supported by the current results, indicating that prior TACE does not have an impact on sequential TARE therapy. The regression analysis found no relationship between the number of prior TACE treatments and outcome in this patient cohort receiving up to seven sessions of chemoembolization.

In particular, the fact that TACE can be repeated several times before employing TARE as a sequential escalating therapy option in the event of tumor progression may be seen as an advantage of initial TACE therapy (15). On average, 60% of HCC patients treated with TACE receive multiple treatment sessions compared with 70% of TARE patients receiving only a single treatment (18, 21–23); in the current study, 73% of patients in the TACE/TARE group received multiple prior chemoembolization. TARE therapy was repeated once in 12 patients: twice in two patients in the TARE-only group and once in eight patients in the TACE/TARE group. The number of TARE sessions is limited by more extensive collateral damage to residual liver tissue during treatment, depending on the type of TARE execution (i.e., lobar vs. segmental).

Although encouraging data are available from smaller retrospective studies regarding combination therapies in large HCC lesions for both TACE and TARE (24–27), the current results support the established concept that the Child–Pugh score and the lesion size (>10 cm) have an effect on the outcome (28); lower OS was found in patients with higher tumor burden and reduced liver function. On the contrary, no differences were found in OS between the TACE/TARE and the TARE-only subgroup. In contrast to previous studies evaluating the impact of tumor radiation dose in TARE, the applied radiation dose did not have an impact on OS in the current study, mostly including patients receiving lobar therapy (29).

The main limitation of this study is the retrospective design with a relatively small, heterogeneous patient cohort with multifocal/advanced HCC. This study was not conceptualized to investigate the therapeutic potential of TARE or TACE with regard to OS. In fact, due to the retrospective nature of this study, patients were included (e.g., portal-venous infiltration) who, according to the current guidelines, are not considered primary candidates for TACE or TARE (30). OS analysis is further limited by an uneven distribution of Child status within Group A and Group B. Thus, the cohort composition may be seen as an explanation for the comparably low OS rates when compared with the current data (31–33). Nonetheless, LTC and PFS did not differ in the cohorts, underlining the technical feasibility of sequencing the procedures.

The tumor-absorbed dose could not be calculated for all patients; therefore, the total applied radiation dose was investigated as a dosimetry parameter. Advanced strategies such as personalized TARE, including radiation segmentectomy/lobectomy (34) or ethanol embolization (35), were not investigated.

## Conclusion

As there are currently few data available on sequential therapy of TACE and TARE, our results provide preliminary evidence that prior TACE does not impair the therapeutic effect of TARE in multifocal, unresectable HCC treatment. However, further studies involving larger and controlled patient cohorts in this area are needed.

## Data Availability

The raw data supporting the conclusions of this article will be made available by the authors, without undue reservation.
